# *In silico* evaluation of the role of Fab glycosylation in cetuximab antibody dynamics

**DOI:** 10.3389/fimmu.2024.1429600

**Published:** 2024-08-08

**Authors:** Simona Saporiti, Davide Bianchi, Omar Ben Mariem, Mara Rossi, Uliano Guerrini, Ivano Eberini, Fabio Centola

**Affiliations:** ^1^ Analytical Excellence and Program Management, Merck Serono S.p.A., Rome, Italy; ^2^ Dipartimento di Scienze Farmacologiche e Biomolecolari, Università degli Studi di Milano, Milan, Italy; ^3^ Dipartimento di Scienze Farmacologiche e Biomolecolari & Data Science Research Center (DSRC), Università degli Studi di Milano, Milan, Italy

**Keywords:** N-glycosylation, Fab, cetuximab, molecular dynamics, ADCC, FcγRIIIA

## Abstract

**Introduction:**

N-glycosylation is a post-translational modification that is highly important for the development of monoclonal antibodies (mAbs), as it regulates their biological activity, particularly in terms of immune effector functions. While typically added at the Fc level, approximately 15-25% of circulating antibodies exhibit glycosylation in the Fab domains as well. To the best of our knowledge, cetuximab (Erbitux^®^) is the only therapeutic antibody presenting Fab glycosylation approved world-wide targeting the epidermal growth factor receptor for the treatment of metastatic-colorectal and head and neck cancers. Additionally, it can trigger antibody-dependent cell cytotoxicity (ADCC), a response that typically is influenced by N-glycosylation at Fc level. However, the role of Fab glycosylation in cetuximab remains poorly understood. Hence, this study aims to investigate the structural role of Fab glycosylation on the conformational behavior of cetuximab.

**Methods:**

The study was performed *in silico* via accelerated molecular dynamics simulations. The commercial cetuximab was compared to its form without Fab glycosylation and structural descriptors were evaluated to establish conformational differences.

**Results:**

The results clearly show a correlation between the Fab glycosylation and structural descriptors that may modulate the conformational freedom of the antibody, potentially affecting Fc effector functions, and suggesting a negative role of Fab glycosylation on the interaction with FcγRIIIa.

**Conclusion:**

Fab glycosylation of cetuximab is the most critical challenge for biosimilar development, but the differences highlighted in this work with respect to its aglycosylated form can improve the knowledge and represent also a great opportunity to develop novel strategies of biotherapeutics.

## 1 Introduction

Immunoglobulins, particularly G1 isotype (IgG1), are the most abundant glycoproteins in circulation and represent one of the most important mechanisms of protection against pathogens (1). They can exert protection through direct interaction with the antigen (2–4) and by recruiting specific receptors, that further activate effector functions such as antibody dependent cell cytotoxicity (ADCC), antibody dependent cell phagocytosis (ADCP), and complement dependent cytotoxicity (CDC) (5–7). IgG1 are often commercialized as monoclonal antibodies (mAbs), potent biotherapeutics used in the treatment against several diseases. From a structural perspective, mAbs are composed of four chains, two light and two heavy (LC and HC, respectively), that are assembled to form a symmetric structure organized in two fragment antigen binding (Fab) domains and one fragment crystallizable (Fc) linked by a flexible hinge region. LCs and HCs are connected by disulphide bonds and contain both variable and constant regions of which the variable ones are involved in the antigen binding (8). The neutralizing effect of mAbs is due to the complementarity determining regions (CDRs), located in the Fab region, that constitute the heterogeneity and uniqueness of the antibody. Moreover, the interaction with immune receptors is determined by the Fc domain, that is very conserved among the different classes of antibodies. A key role in modulating the interaction with Fc receptors is played by the N-glycosylation, a post-translational modification that occurs at the conserved Asn297 in the Fc region (9, 10). It is well known that differences in the N-glycosylation pattern, such as the presence of fucose, can modulate the Fc receptors recognition (11, 12) and several published studies demonstrated a role of this modification not only in modulating the bioactivity of the mAbs (13–20), but also their conformational behavior (21, 22).

Although Fc glycosylation is always present on IgGs and represents an important aspect to be considered during product development and in the post-marketing surveillance, N-glycosylation in the Fab region also needs to be evaluated. Actually, Fab glycosylation has been observed in approximately 15-25% of all circulating antibodies and it is associated with many physiological and pathological conditions (23): *e.g.*, in pregnancy, where Fab-glycosylated IgG antibodies from the mother are directed against paternal antigens (24); in auto-immune diseases like rheumatoid arthritis (25); in primary Sjogren’s syndrome (26); and in B cell malignancies (27–29).

A prerequisite to have a N-glycosylated site in both Fab and Fc is the presence of the N-X-S/T sequence motif, where X is not Pro (9). This condition is however not sufficient to observe the Fab glycosylation, that can happen in both LC and HC, in CDRs, and in framework regions, because it is due to a somatic hypermutation during Ag-specific immune responses (30) and for biotherapeutics it can depend on the chosen germline. For instance, mouse IGKV5-45 allele presents a germline-encoded glycosylation site that is not occupied in both infliximab (31) and cetuximab (32, 33), two therapeutic commercial mAbs. However, the cetuximab produced via mouse IGHV2-2 germline is glycosylated at the same site (32–34). Many factors can also influence Fab glycosylation patterns, including the position (35, 36) and the proximal amino acids (37–39). Even if a certain population of high mannose species has been detected, the most frequent population of Fab glycosylation is based on a biantennary species, which often has one or two terminal α2,6-linked sialic acids, with a pattern that is very similar to the one in the Fc region (40). Several studies report that Fab glycosylation can increase or decrease Ag binding, block binding between two proteins by steric hindrance, extend Ab half-life because of sialylation, and presumably affect Ab aggregation and immune complex formation (23). In the context of biotherapeutic mAbs and to the best of our knowledge, cetuximab, marketed as Erbitux^®^, is the only antibody presenting a Fab glycosylation approved world-wide. Targeting the epidermal growth factor receptor (EGFR), it is used for the treatment of metastatic-colorectal cancer and head and neck cancer (41, 42). Cetuximab is a chimeric mouse/human recombinant antibody produced in SP2/0 mouse myeloma cell line (42), presenting a wide structural heterogeneity due to a second non-human N-glycosylation site located at Asn88 within the framework 3 of the variable domain of HC (Fab portion), in addition to the classical site at Fc (Asn299) (43). The functional role of Fab glycosylation in cetuximab is still not clear. It seems to not affect the binding to the antigen because it is on the opposite site with respect to CDRs (33). Moreover, Giddens et al. showed that glycoengineered cetuximab uniformly carrying single fully sialylated Fab N-glycans exhibits the same affinity as commercial cetuximab carrying multiple N-glycans (44). On the other hand, the pharmacokinetic profile of cetuximab can be influenced by Fab properties, *i.e.*, charge variants and N-glycosylation, as demonstrated by Schlothauer and colleagues who observed an increase of affinity between Fc and neonatal Fc receptor (FcRn) after the removal of Fab domains (45). As aforementioned, cetuximab is produced in mouse cells and N-glycans show a high heterogeneity. This aspect is linked to severe hypersensitivity reactions in some patients, especially because the 30% of glycans in the Fab contain one or two α1,3-galactose and a significant content of N-glycolylneuraminic acid (Neu5Gc) that are both considered highly immunogenic (43, 46). Cetuximab is also known to activate the ADCC function, which is claimed as secondary mode of action of the molecule (47–49) and that may be influenced by the surface charge of the antibody, since different charge variants can influence the affinity to FcγRIIIa (50). As already reported in our previous works, N-glycosylation at Fc domain can have not only an impact on FcγRIIIa recognition and ADCC activation, but also on the antibody conformational behavior, influencing the preference of the antibody for certain conformational states (21, 22). Accordingly, the scope of this work, that was entirely developed *in silico*, is to investigate via computational tools the role of Fab glycosylation on cetuximab dynamics, trying to shed light on its mechanism of action. Accelerated molecular dynamics (aMD) simulations were performed to compare the commercial cetuximab glycosylated at Fab and its aglycosylated form to evaluate the structural descriptors that can modulate the conformational freedom of the antibody, potentially affecting the Fc effector functions. This potential role of Fab glycosylation has never been investigated before and can be a focus for the development of a new class of biotherapeutic mAbs, where the modulation of the effector functions is performed throughout the Fab glycosylation.

## 2 Materials and methods

### 2.1 Homology modeling

The three-dimensional (3D) structure of cetuximab was built with a chimeric homology modeling approach by the “Homology model” tool of MOE 2022.02 software (51). The X-ray structure of cetuximab Fab was used to model these domains (PDB ID: 1YY8) (32), while the structure of a fully human IgG1, contained in MOE library of crystalized antibodies was used as a template (PDB ID: 1HZH) (52) to build hinge and Fc portion. Before performing homology modeling, all the templates were prepared using the “Structure Preparation” tool of MOE, to correct any crystallographic issue, and processed by the “Protonate 3D” tool to assign the ionization states and add missing hydrogens. 1YY8 structure presents some missed residues at the C-terminal of LC, namely Glu213 and Cys214 that were modeled on 1HZH with the enabled “override template” option. The homology modeling procedure generated 10 intermediate models, and the final structure was chosen based on the highest-scoring intermediate model. The score was determined using the generalized Born-volume integral methodology, which calculates the free energy of hydration by summing the electrostatic energy term with a cavitation energy derived from a volume integral London dispersion energy (53). An energy minimization step was executed until the root mean-square (RMS) gradient reached a value of 0.5 kcal/mol/Å^2^, opting for the “medium” setting to facilitate a moderate relaxation and alleviate steric strain. The model was then glycosylated at the conserved Asn297 in the Fc (Asn299 in cetuximab) with G0F glycans obtained from 1HZH structure, linking the ND2 of the Asn and the C1 of N-acetylglucosamine (GlcNac). To obtain the cetuximab model glycosylated at Fab, G2F+2αGal glycans were attached unit by unit to Asn88 in the HC and then the model was again energy minimized until the RMS gradient of 0.01 kcal/mol/Å^2^. G2F+2αGal on Fab domains and G0F on Fc are reported in literature as the most expressed in the commercial antibody produced in SP2/0 cells (43) and for this reason they were considered in this study.

### 2.2 Classical and accelerated MD simulations protocol

aMD simulations of cetuximab and cetuximab without Fab glycosylation were performed. aMD is a powerful tool able to simulate infrequent events that are required for protein conformational changes applying a bias potential that forces the system to overcome potential energy barriers (54). This method can be used without previous knowledge of the conformational states or, oppositely than metadynamics, without collective variables, *a priori* defined. Among many applications, aMD has been used to predict peptide folding (55), investigate protein-ligand interactions (56), analyze the behavior of viruses envelope (57), and also to predict the conformational behavior of commercial mAbs, describing the role of N-glycosylation and LC isotype in the IgG1 dynamics (22).

Before running aMD simulations, classical MD (cMD) was necessary to obtain input parameters for the aMD. For both cMD and aMD simulations, the systems were prepared using the CHARMM-GUI webserver (58) and were solvated in a cubic water box with dimensions of 181 Å × 3, ensuring a minimum edge distance of 15 Å and adding NaCl 0.15 M to neutralize the charge. Simulations were run by AMBER20 (59), using CHARMM36 forcefield (60) for solute parametrization and TIP3P water model for solvent. For energy minimization, a steepest-descent algorithm was applied for 5000 cycles, with positional restraints on the protein and sugars, along with dihedral restraints on the sugars. The equilibration phase was run for 125 ps with a time step of 0.001 ps in the NVT ensemble at T = 300 K with the SHAKE algorithm for constraining hydrogen atom vibrations, and the particle mesh Ewald (PME) method (61) with a cutoff value of 12 Å for calculating electrostatic interactions. One cMD simulation 50 ns long was performed for each model (cetuximab and aglycosylated cetuximab) with the scope to obtain the average potential energy (EPTOT) and the average dihedral angle energy (DIHED), that were used as input for aMD. The production phase of these cMD simulations was performed in NPT ensemble (T = 300 K, P = 1 bar) with Langevin thermostat and Monte Carlo barostat. The sample time was set to 0.002 ps and saving energies every 1,000 steps and coordinates every 5000 steps. Additionally, minimized and equilibrated input systems were used for the production phase of aMD that was performed for 1 µs saving energy and coordinates every 100 ps and using a time step of 0.002 ps. The NPT ensemble (T = 300 K; P = 1 bar) with a Langevin dynamics for the temperature control and Berendsen barostat for pressure, and a whole potential boost together with an extra boost to the torsions (*iamd* = *3*) were applied. The equations used to calculate the values of EthreshD, EthreshP, alphaD and alphaP, that are necessary for aMD, are reported in our previous work (22).

### 2.3 Analysis of trajectories

The motion of Fab domains was characterized using *ϕ* (longitude) and *θ* (latitude) angles within a reference frame attached to Fc and centered at the hinge. The axes were defined as follows: the z-axis aligned with Fc and directed toward Fab domains, the x-axis parallel to a vector connecting the mid-Fc (CH2 regions), and the y-axis determined by the right-hand rule. For a comprehensive explanation, please refer to our previous study (21). A reweighting procedure was applied according to methods described by Miao et al. (62) using Maclaurin expansion to the 10^th^ order to approximate the free energy surface (FES) of the system as a function of *θ* angles. Once identified the minimum energy region of the FES, all the subsequent analyses were performed for those frames, namely 1652 frames for cetuximab and 2271 frames for cetuximab without Fab glycosylation. The root mean square deviation (RMSD) matrices for the cluster analysis were obtained with CPPTRAJ (63), while the clusters were obtained using a customized script based on the GROMOS algorithm (64), considering Cα atoms and a RMSD-threshold of 6.5 Å. The maximum number of clusters was set to 10. *Φ* angle variation was computed per each Fab and the *Δϕ* distribution was plotted according to Equation 1:


(1) $$\text{Δφ}=\varphi_{i}-\varphi_{0}$$

The angle between Fab domains was computed by the “angle” tool available in CPPTRAJ (63), considering the Cα atom of Val34 in CDRH1, the SG atom of Cys228 in the hinge and the Cα atom of Val34 in the second HC. The contacts between LCs and the hinge region were computed by CPPTRAJ (63) with the “nativecontacts” tool, considering heavy atoms and a threshold distance of 4 Å. The hydrogen bonds (H-bonds) analysis was computed by a customized python script based on the MDTraj H-bonds identification tool (65) and considering a cutoff frequency value of 1%. The CH2 distance was computed between glycosylated Asn299 in the Fc by MDTraj (65). The minimum distance between glycan chains was computed by CPPTRAJ (63) and the “nativecontacts” tool with the “mindist” option. The secondary structure content was computed by the “secstruct” tool of CPPTRAJ according to the DSSP algorithm (66). The correlation matrix was obtained by calculating the covariance matrices of atoms fluctuation along the minimum energy frames of aMD simulations. The covariance matrix was then normalized by the standard deviation. This correlation matrix effectively represents the interdependent relationships between the amount of movement (fluctuation) of all protein residues. The solvent accessible surface area (SASA) was calculated via the “surf” tool by CPPTRAJ (63), while protein patches were evaluated by MOE 2022.02 with the “Surfaces and Maps” tool (51).

### 2.4 Statistical significance

Statistical significance of the differences between the distributions of Fab-sugars contacts, SASA of hinge, FcγRIIIA and FcRn binding residues, and the angle between Fab domains, was computed using the Student’s t-test and expressing the refusal of the null-hypothesis in terms of *p*-value. Due to the large sample size, following the work by Lee (67), we also reported the effect size in form of 95% confidence interval of Cohen’s *d*. As usual we considered small effect *d* ~ 0.2, medium effect *d* ~ 0.5, and large effect *d* ~ 0.8 and above. All the calculations were performed via an in-house python script using SciPy and Pandas libraries (68).

## 3 Results

### 3.1 Three-dimensional structure of cetuximab

The 3D structure of cetuximab was obtained via homology modeling as described in “Materials and methods” section. Two models were generated, both glycosylated at the Fc portion with G0F glycans (Asn299 in HC), but only one glycosylated also at Fab domain with G2F+2αGal pattern (Asn88 in HC). The structures are shown in **Supplementary Figure 1** with the corresponding Ramachandran plot and a 2D representation of N-glycans according to the Symbol Nomenclature For Glycans (SNFG) scheme (69). As shown, very few outliers are present and all localized in unstructured regions. These models were submitted first to a cMD simulation 50 ns long to obtain the values of EPTOT and EDIHED needed for the following aMD simulations. The values of these parameters were used to compute EthreshD, EthreshP, alphaD and alphaP that are reported in **Supplementary Table 1**.

### 3.2 Analysis of Fab behavior

#### 3.2.1 Free energy surface along θ angles

**Figure 1:**
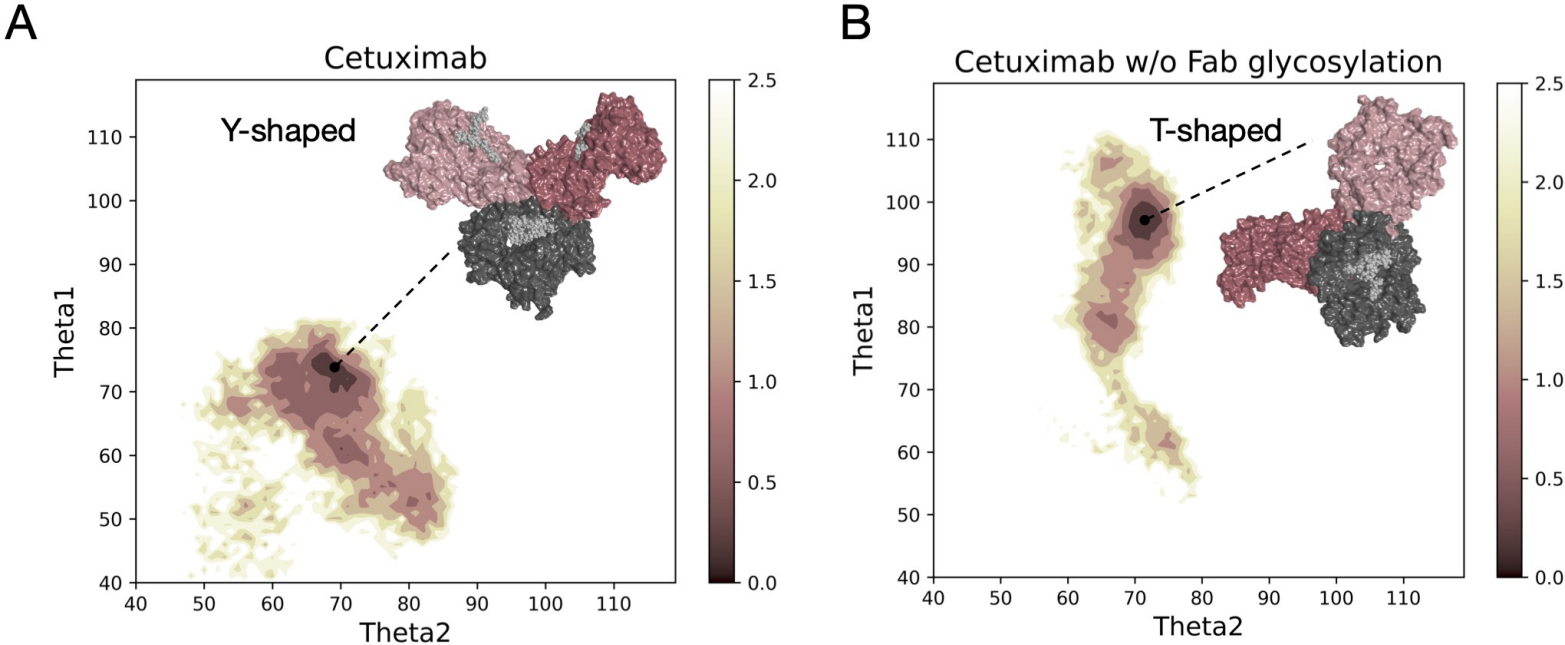
Free energy surface of cetuximab and cetuximab without glycosylated Fab along θ angles. The reweighted free energy profile of cetuximab **(A)** and cetuximab without Fab glycosylation **(B)** along Theta1 and Theta2 angles. The molecular surface of medoid structures isolated from the minimum energy region (in dark pink) by cluster analysis is also shown. The color bar represents the PMF value in kcal/mol.

Both models, cetuximab and cetuximab without Fab glycosylation, were submitted to aMD simulations 1 µs long to find an energy minimum corresponding to a stable conformation. The FES of the two antibodies was plotted along *θ* angles, Theta1 and Theta2, that describe the position of Fab1 and Fab2 domains with respect to Fc. This descriptor allows the classification of antibody conformations in two categories: Y-shaped and T-shaped. Y-shaped forms present θ values < 90°for both Fab domains, while T-shaped conformations show θ ≥ 90° in at least one Fab (22). Accordingly, in **Figure 1** the FES profile of both cetuximab forms is reported, showing not only a different exploration in terms of potential energy surface, but also two different conformations, isolated by cluster analysis in the corresponding energy minimum region (**Supplementary Figure 2**). Specifically, cetuximab presents a Y-shaped conformation while cetuximab without Fab glycosylation presents a form that can be approximately described as T-shaped. The minimum energy region corresponds to the value of potential of mean force (PMF) < 0.5 kcal/mol. Herein, cetuximab presents both Theta angles ≈ 70°, while the aglycosylated form shows Theta1 ≈ 95° and Theta2 ≈ 70°. Considering this result, a role of Fab glycosylation in driving the conformational behavior of cetuximab can be hypothesized and the following analyses were performed in the minimum energy frames thus identified.

#### 3.2.2 Description of Fab rotation

**Figure 2:**
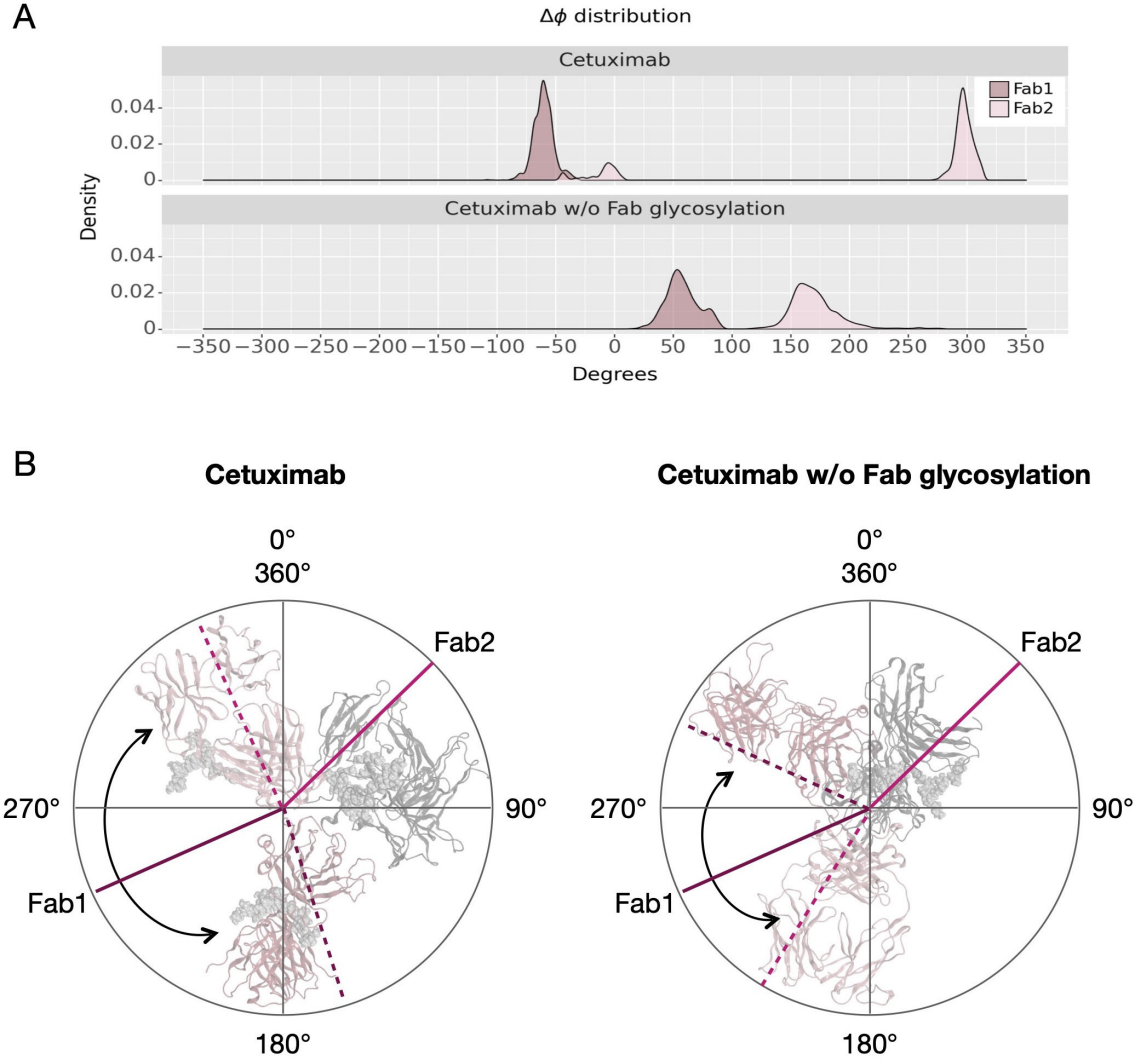
*Δϕ* distribution and schematic representation of Fab rotation. **(A)** *Δϕ* distribution of cetuximab and cetuximab without Fab glycosylation. **(B)** Schematic representation of the change of *ϕ* angles from the starting position (continuous lines) to the minimum energy conformation (dashed lines). Lines corresponding to Fab1 are shown in purple, while those corresponding to Fab2 in pink. Black arrows indicate the angle between two Fab domains.

**Figure 3:**
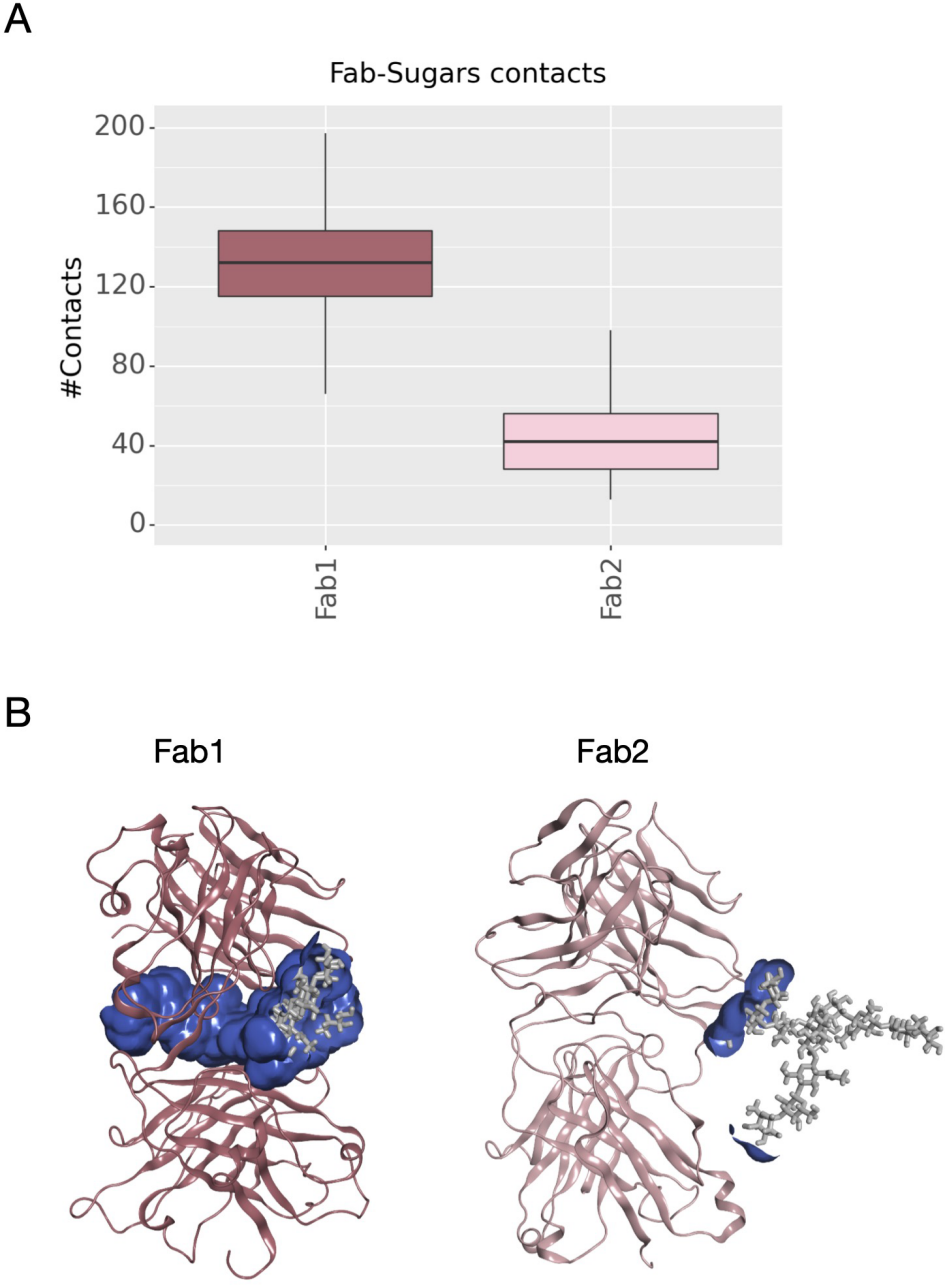
Number of contacts between N-glycans and Fab domains and structural representation of the interaction surface. **(A)** The box plot showing the distribution of contacts number between Asn88 glycans and Fab domains. **(B)** The structural representation of Fab domains as ribbons and the interaction surface (in blue) between the protein and sugars. N-glycans are shown as light gray sticks.

**Figure 4:**
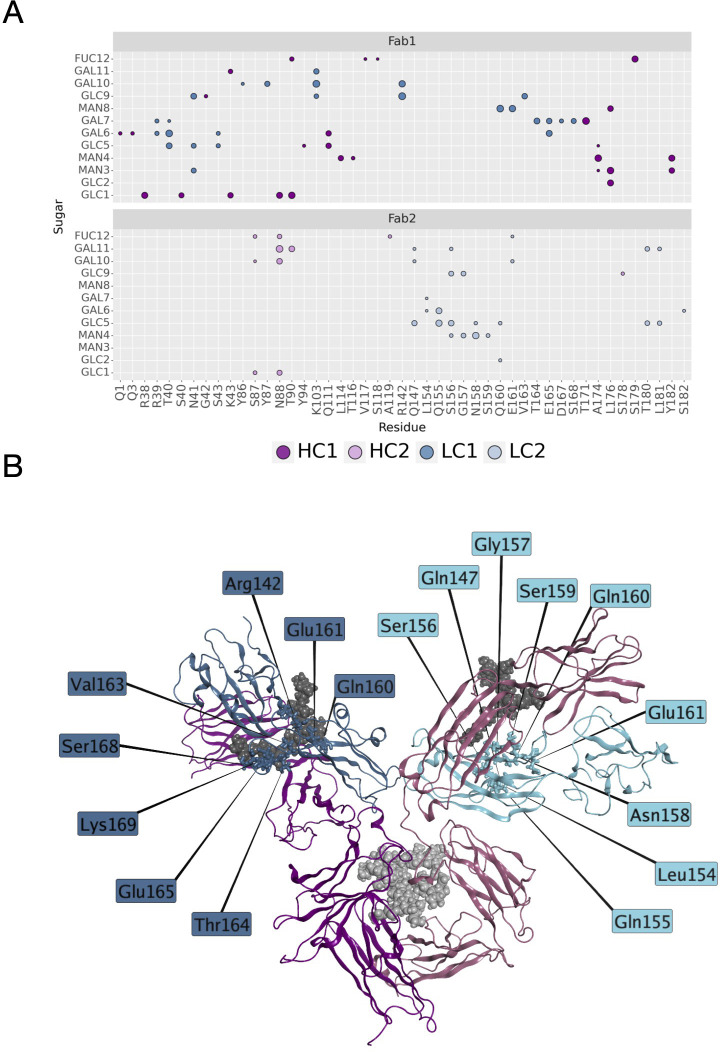
H-bonds between N-glycans and Fab domains. **(A)** A matrix showing the H-bonds between sugars and mAb colored by chain. The size of the markers represents the frequency of the H-bond in the minimum energy frames: low size corresponds to low frequency, high size to high frequency. **(B)** Structural representation of cetuximab in the minimum energy conformation with the LC residues involved in the interaction with sugars. The antibody is represented as ribbons colored by chain (in blue and light blue, LC1 and LC2, respectively; in dark magenta and pink, HC1 and HC2, respectively). N-glycans are shown as spheres, while LC residues as sticks.

**Figure 5:**
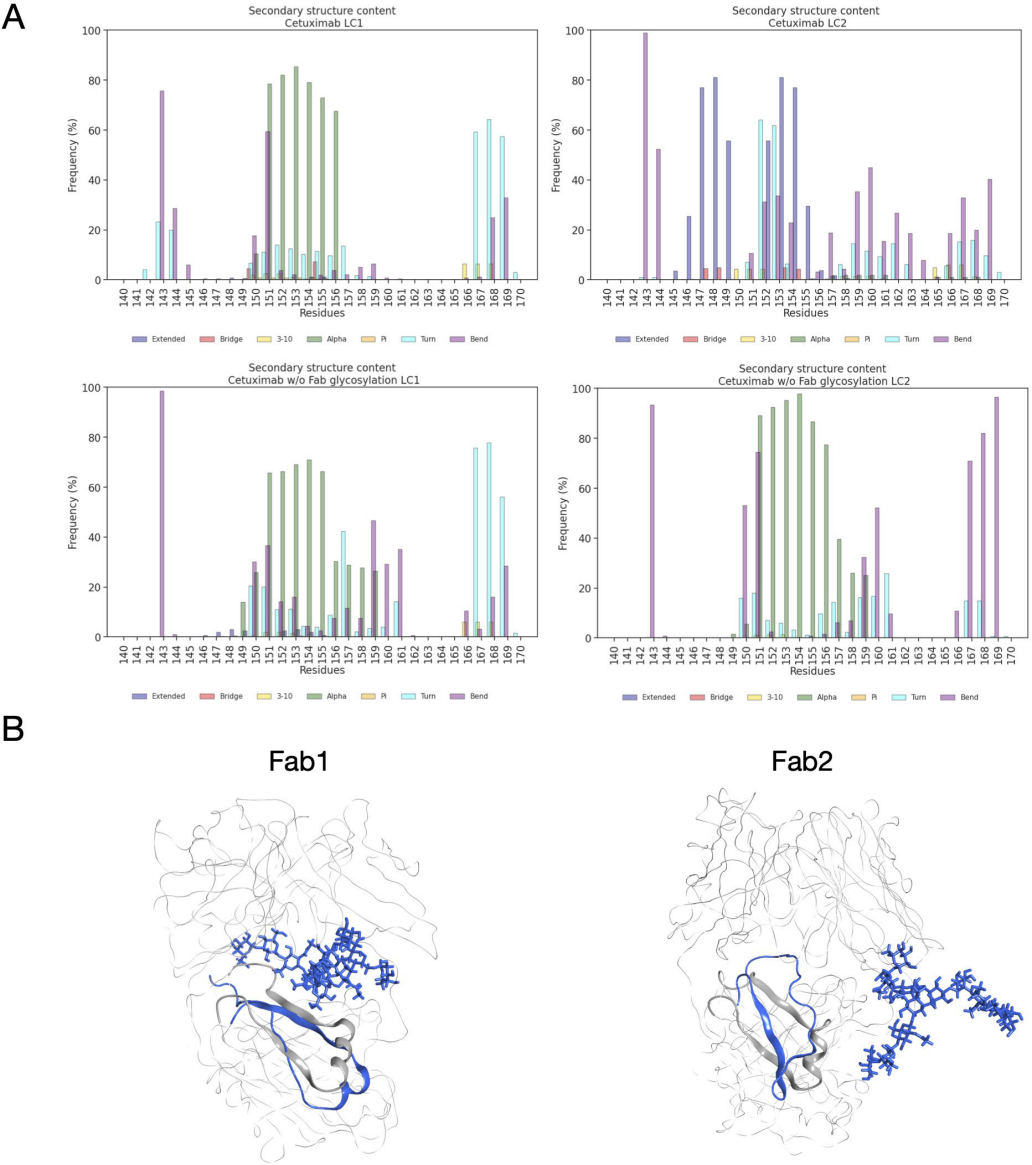
Secondary structure content of LC peptide in cetuximab forms. **(A)** Bar plots showing the per residue secondary structure content in the LC peptide (res. 140-170) of cetuximab (top) and cetuximab without Fab glycosylation (bottom). The frequency of each secondary structure type according to DSSP classification is reported in percent on y-axis. **(B)** Structural superposition of glycosylated (in blue) and aglycosylated (in gray) Fab domains showing the differences in the secondary structure of LC peptides.

Another descriptor useful to investigate the behavior of Fab domains is the *ϕ* angle. This angle is representative of Fab rotation and can define the flexibility of this domain considering movements in a different direction than *θ* angle. To observe the shift of Fab position with respect to the starting frame, *Δϕ* distribution (Eq. 1) was plotted (**Figure 2**). Accordingly, as already observed in our previous work (21), Fab domains move asymmetrically, with Fab2 rotating more than Fab1 in both cetuximab with and without Fab glycosylation. Between the two models, cetuximab with glycosylated Fab domains shows the highest difference in terms of their rotational propensity, with *Δϕ* values around -70° for Fab1 and 300°for Fab2. In **Figure 2A**, *Δϕ* distribution for each Fab domain in each cetuximab model is reported, together with a graphical representation of the movement that Fab domains perform with respect to their starting position (**Figure 2B**). The graph shows that the angle between Fabs depends on the species, with aglycosylated Fab domains having a median angle of 93.2° and the glycosylated one of 107.1°, compared to the starting angle of 88.1°, suggesting that this parameter can influence the final conformation of the antibody, too. In **Supplementary Figure 3**, the boxplot showing the distribution of Fab angles is reported. To explain the different rotational propensity of glycosylated Fab domains, an investigation of the interaction network between Fab glycans and the antibody was carried out. The number of contacts between glycans and Fab was computed, showing that Fab1 is involved in much more interactions than Fab2 (**Figure 3A**). This result suggests both the role of N-glycans linked to Asn88 in stabilizing the movements of this domain and the asymmetric behavior typical of mAbs (**Figure 3B**). The analysis of H-bonds network between Fab and sugars shows that there is a specific peptide in LC that is involved in the interaction with glycans (**Figure 4**). This peptide comprises the region from Tyr140 to Asp170 and its interaction network with sugars is less stable for LC2 than for LC1, as suggested by the lower frequency values of the H-bonds (**Supplementary Table 2**). This asymmetric behavior can explain the different rotational propensity of the two Fab domains, supporting the analysis of contacts number and suggesting a direct structural impact of glycosylation on LC behavior. Accordingly, the secondary structure content of this LC peptide was evaluated in both cetuximab and in the cetuximab without Fab glycosylation showing not only a difference between LC1 and LC2 in the glycosylated form, but also between the two simulated models. As reported in **Figure 5A**, the presence of N-glycosylation in Fab can induce a rearrangement of the secondary structure of this peptide that, with respect to the aglycosylated form, loses its typical α-helix motif in the region between res. 151-159 in favor of unstructured amino acids (*i.e.*, bend, turn, or no structure). In LC2, there is a complete change of the structure in this region, that from α-helix is converted in extended β-sheets, bend or turn. In **Figure 5B** the structural representation of Fab domains is reported highlighting also the differences in the LC peptide between the two cetuximab forms. Since the structural proximity of this region to res. 161-191 in HC, also the secondary structure content of this region was evaluated (**Supplementary Figure 4**). As a result, no big impact was observed, suggesting a stronger effect of Fab glycosylation on LC than on HC. The statistical significance of the observed differences in Fab-sugars contacts and the angle between Fab domains is reported in **Supplementary Material** (“Statistical significance results” section) and in **Supplementary Table 3**.

### 3.3 Analysis of Fc behavior

**Figure 6:**
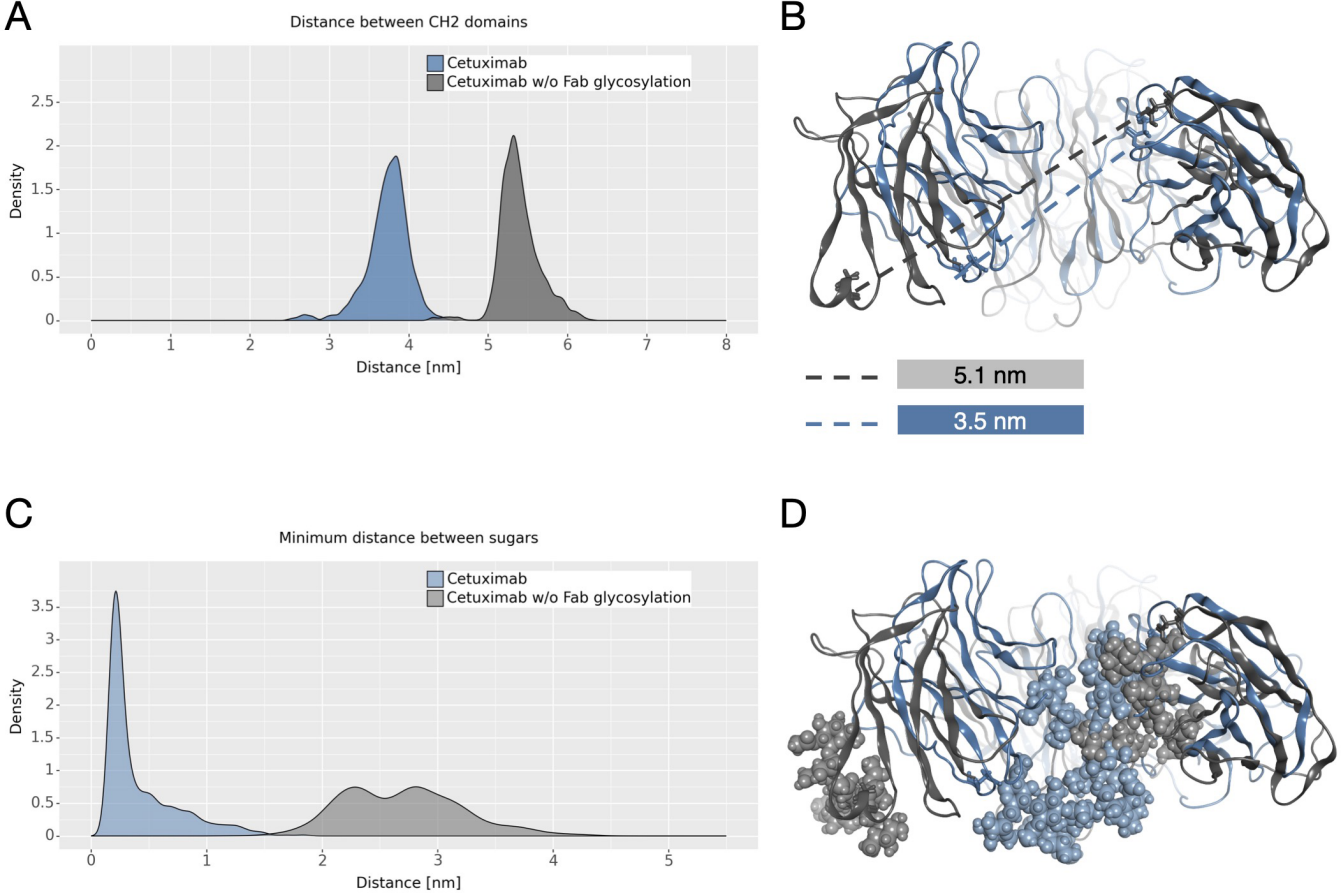
CH2 distance and minimum distance between Fc sugar chains. **(A)** The density plot of the distance between CH2 domains of Fc. **(B)** The structural superposition of Fc domains of cetuximab (in steelblue) and cetuximab without Fab glycosylation (in gray). The CH2 distance in the minimum energy structure is also reported. **(C)** The density plot of the minimum distance between the center of mass of Fc sugar chains. **(D)** The structural superposition of Fc domains of cetuximab (in light steelblue) and cetuximab without Fab glycosylation (in light gray) showing the position of sugars in the Fc cavity in the minimum energy structure.

The dynamical behavior of Fc fragment was investigated according to two descriptors previously identified (21, 22). The first one is the distance between CH2 domains that is an indicator of Fc opening. According to literature, larger CH2 distance corresponds to open Fc domains and to higher affinity for FcγRIIIa (70). As shown in **Figure 6A** the two antibodies present different distributions of this distance, with higher values in cetuximab without Fab glycosylation. This suggests that the presence of glycosylation in the Fab domains could potentially lead to close Fc conformations that are less prone to interact with the receptor (**Figure 6B**). The second descriptor is the distance between the center of mass of sugar chains inside the Fc cavity (**Figure 6C**). As previously observed, fucosylated sugars prefer to stay into the Fc hydrophobic pocket more than afucosylated ones (21, 22) suggesting that the lower is this distance the less is the affinity of the antibody for FcγRIIIa. In this case, even if this distance is very small for both systems, cetuximab without Fab glycosylation presents slightly higher values, suggesting that the abrogation of Fab glycosylation can also influence the behavior of sugars at the Fc level, in principle favoring conformations more prone to interact with the receptor (**Figure 6D**).

### 3.4 Covariance analysis

**Figure 7:**
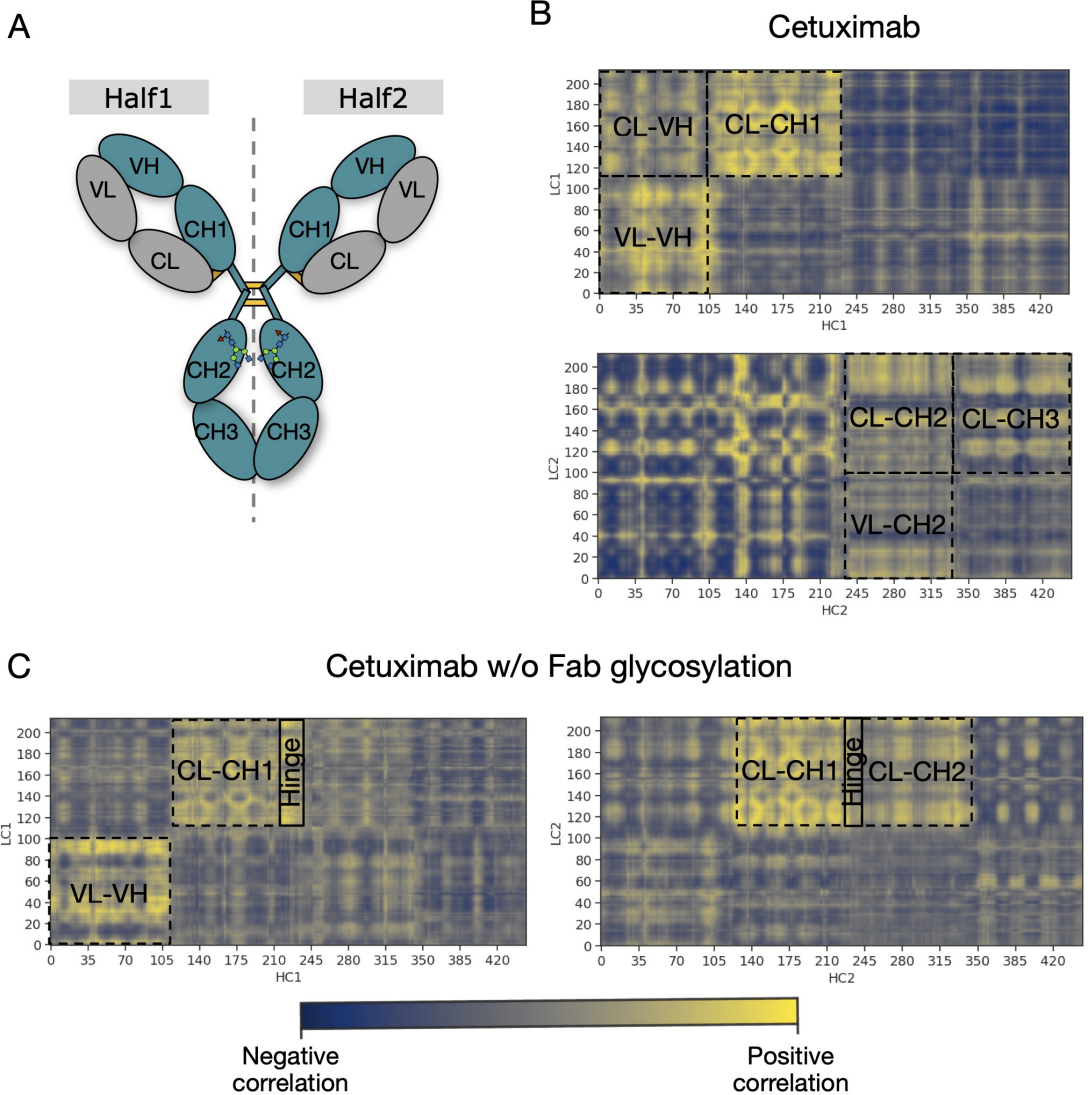
Inter-chain correlations in cetuximab and cetuximab without Fab glycosylation. **(A)** Schematic representation of a classical IgG1 architecture indicating single domains of each chain. Correlation matrices between LC and HC for each half of cetuximab **(B)** and cetuximab without Fab glycosylation **(C)**. Black squares highlight strong positive correlations (in yellow) between domains.

**Figure 8:**
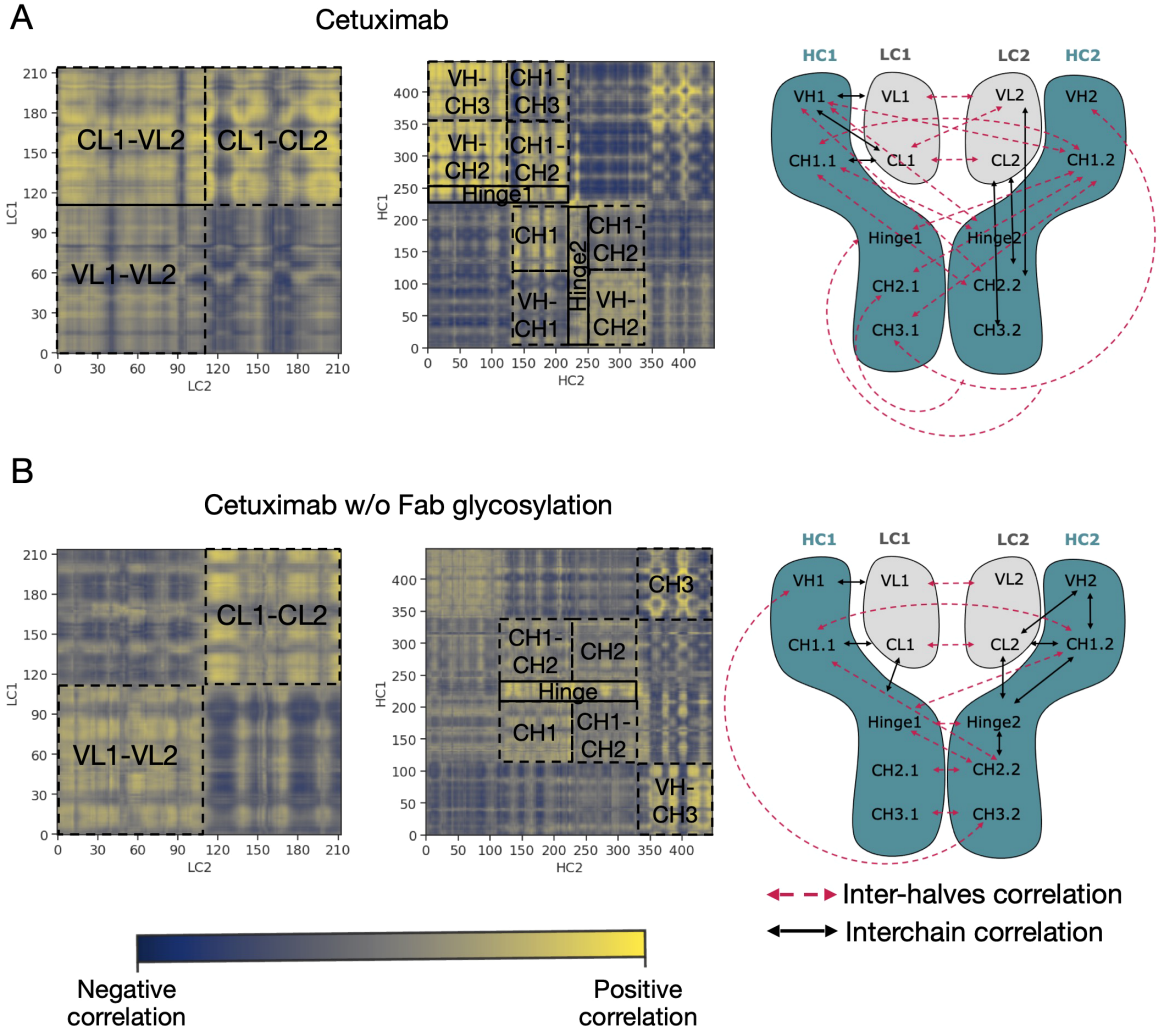
Inter-halves correlations in cetuximab and cetuximab without Fab glycosylation. Correlation matrices between corresponding LC and HC belonging to the different antibody halves of cetuximab **(A)** and cetuximab without Fab glycosylation **(B)**. Black squares highlight strong positive correlations (in yellow) between domains. A schematic representation of inter-chain and inter-halves correlations in the two antibodies is reported alongside the correlation matrices.

An analysis of covariance based on the fluctuation of Cα atoms was carried out to identify the independent correlations among the movements of antibody residues. According to the results, several positive correlations were identified between structured domains both in cetuximab and in cetuximab without Fab glycosylation. Considering the symmetric structure of the mAb, the correlations were classified in inter-chain and inter-halves. Inter-chain correlations refer to the positive correlations between LC and HC belonging to the same half of the antibody, while the inter-halves correlations are those occurring between chains included in different halves (**Figure 7A**). Regarding the inter-chain correlations, as summarized in **Figures 7B, C**, in both cases they involve regions that are in structural proximity, *i.e.*, VH-VL and CL-CH1. However long-distance correlations were also observed between CL-VH, VL-CH2, CL-CH2 and CL-CH3 in cetuximab, and between CL-Hinge and CL-CH2 in cetuximab without Fab glycosylation. Considering the inter-halves correlations, they are more complex and evident in commercial cetuximab (**Figure 8A**) than in the one without Fab glycosylation (**Figure 8B**). Specifically, looking at LC correlations, they are quite conserved between VL1-VL2 and CL1-CL2, while the correlation between CL1-VL2 is present only in the commercial mAb. Regarding HC, much more correlations among domains are present in cetuximab than in the form aglycosylated at Fab domains, suggesting a direct influence of VH domains behavior on Fc and hinge. VH domains include N-glycosylation at Asn88 site, and this let to hypothesize an allosteric effect of this PTM on the general behavior of the mAb and on its propensity to recognize FcγRIIIa. Globally, the correlation network of the commercial mAb is more complicated than that observed in the aglycosylated antibody, suggesting that Fab glycosylation can generate structural constraints in the whole molecule, inducing a specific conformation.

### 3.5 Evaluation of FcγRIIIa and FcRn binding sites behavior

**Figure 9:**
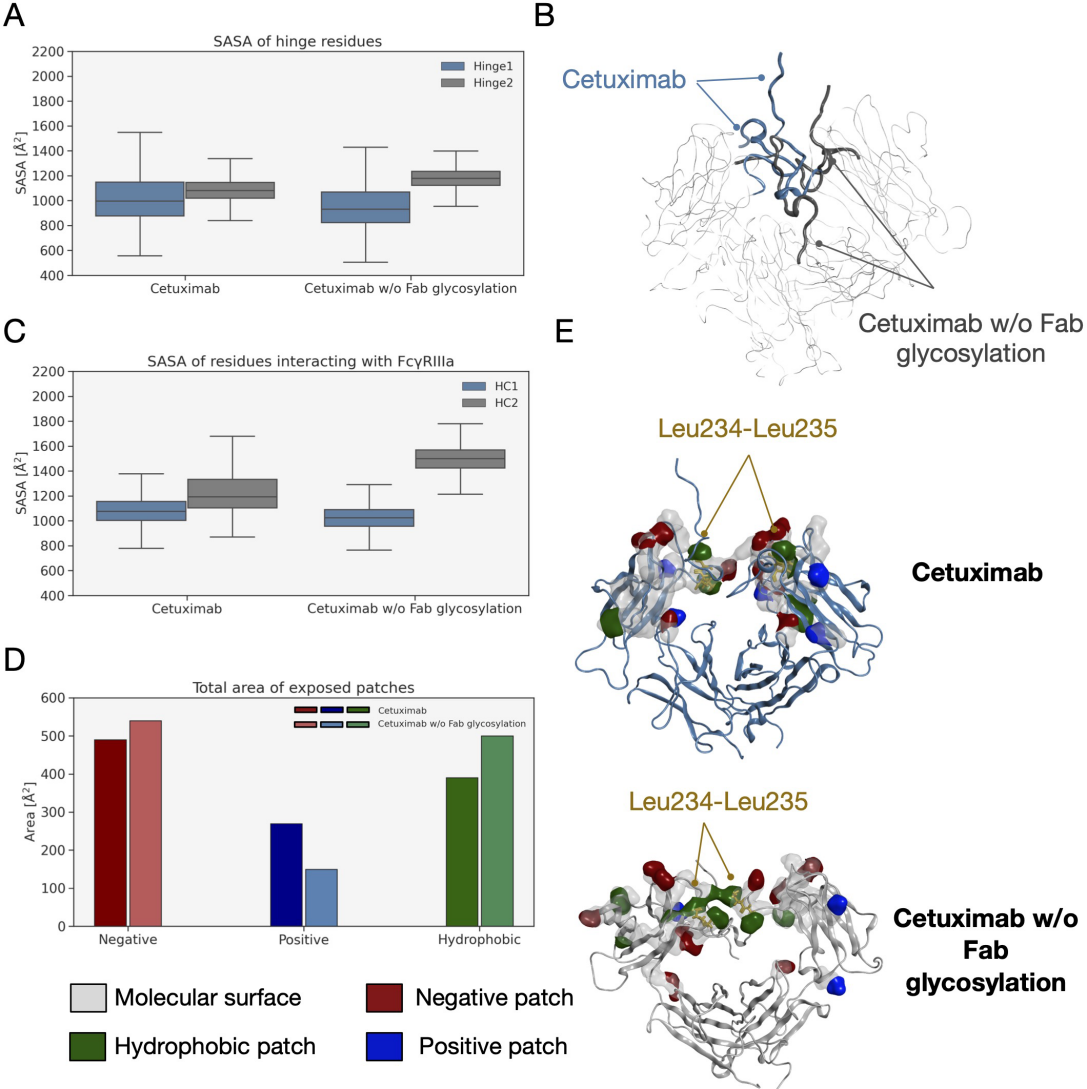
SASA of hinge and FcγRIIIA binding site residues and exposed patches analysis. **(A)** Boxplot showing the distribution of SASA values for hinge. **(B)** Structural representation of hinge orientation in cetuximab and cetuximab w/o Fab glycosylation after Fc superposition. **(C)** Boxplot showing the distribution of SASA values for FcγRIIIa binding residues in the two forms. **(D)** Analysis of protein patches in the minimum energy structures with a bar plot showing the total area of exposed patches by type. **(E)** The structural representation of protein patches surface with the position of Leu234 and Leu235 highlighted in the two models. For clarity, only hinge and Fc are shown as ribbons.

An analysis of the structural behavior of hinge and those residues involved in the interaction with FcγRIIIa was performed to evaluate possible effects of Fab glycosylation on the ability of the antibody to bind the receptor and consequently activate ADCC response. Hinge residues were selected according to IMGT convention (71), while residues involved in the receptor binding were selected according to the work by Shields and colleagues (72). The SASA of these regions was computed showing that both in the case of the hinge and in that of FcγRIIIa binding site, cetuximab without Fab glycosylation presents a higher solvent exposure, suggesting a higher propensity to interact with the receptor than the commercial form (**Figures 9A–C**). Also, in this case the asymmetric behavior typical of mAbs was observed since the main differences can be seen for Hinge2 and HC2. Furthermore, charged and hydrophobic patches on the binding site were evaluated in the minimum energy conformations, showing that the type of exposed surface can change between the two forms. In **Figure 9D**, the total amount of patches area is reported for both cetuximab forms, showing that - in the absence of Fab glycosylation - the total area of negative and hydrophobic patches increases, whereas that of positive patches decreases. **Figure 9E** shows how in particular the hydrophobic patches determined by Leu234 and Leu235 residues are less exposed in the commercial cetuximab. It is well known that these two Leu residues are critical for receptor recognition since mutagenesis analysis showed a reduction of receptor binding when they are mutated in Ala (“LALA” mutation) (73). Overall, these data suggest that Fab glycosylation can have a negative structural impact on the interaction with the receptor and points again out the key role of this modification in modulating the antibody behavior. In addition, an evaluation of the effect of Fab glycosylation on the pharmacokinetics of the antibody, considering the potential binding to FcRn, was performed. Specifically, the SASA of residues involved in the binding to FcRn, selected based on literature data (74–76), was computed and it is reported in **Supplementary Figure 5** together with a structural representation of the binding site in the two conditions. According to these data, the negative impact of Fab glycosylation on the behavior of Fc region is confirmed again. Also in this case, in fact, a higher solvent exposure of the FcRn binding residues is observed in cetuximab without Fab glycosylation (particularly on HC2) than the commercial mAb. This corresponds also to a different structural orientation of the A-B turn in CH2 domain that is more exposed and probably more prone to interact with the receptor. The statistical significance of the observed differences in these data is reported in **Supplementary Material** (“Statistical significance results” section) and in **Supplementary Table 3**.

## 4 Discussion

The scope of this study was to evaluate the impact of Fab glycosylation on the conformational behavior of cetuximab (Erbitux^®^) that is used against metastatic-colorectal and head and neck cancer (41, 42). The potential structural role of Fab glycosylation in modulating immune effector functions was never investigated before, and cetuximab was chosen as a case study because it is the only mAb glycosylated at Fab currently on the market. The study was entirely developed *in silico*, applying aMD to the commercial cetuximab and to its form without Fab glycosylation to find out minimum energy conformations of the two species. The results clearly show a role of Fab glycans in modulating the conformation of the antibody because, two different conformations were found: the commercial antibody presents a Y-shaped conformation, while the cetuximab without Fab glycosylation a T-shaped like form. The T-shaped conformation has been already observed for classical IgG1 presenting fucosylated N-glycans at Fc level (22) and, for this reason, this result was expected. On the other hand, the observation of a Y-shaped conformation even in presence of fucosylated Fc glycans suggests that Fab glycosylation can modulate the dynamics of the mAb. In addition, glycosylated Fab domains are influenced by the glycans in terms of rotational propensity and secondary structure content, especially at the LC (e.g., res. 140-170). Considering Fc, a more open Fc conformation in cetuximab without Fab glycosylation with respect to commercial mAb was observed. According to literature data, opened Fc structures are more prone to interact with the receptor and to activate ADCC response (70). Therefore our results suggest a negative impact of this Fab glycosylation on the ability of Fc to interact with FcγRIIIa. Considering all these aspects, a correlation between the presence of Fab glycosylation and the ability of cetuximab to bind the Fc receptor, activating ADCC cascade, was hypothesized. An analysis of long-distance correlations among domains was performed showing that in cetuximab without Fab glycosylation there is a prevalence of linear correlations, likely corresponding to a more flexible behavior of the protein. On the other hand, in the commercial mAb, the correlations are transversal and even more complicated, occurring between variable domains, especially the glycosylated VH ones, and the hinge and Fc portion. This result, together with the observation of a blocked Y-shaped conformation, may suggest the presence of structural constraints induced by Fab glycans. Finally, a higher solvent exposure of hinge residues and of those residues involved in the FcγRIIIa recognition was observed in the case of cetuximab without Fab glycosylation, suggesting a higher propensity of this form to interact with the receptor. Looking at the structure, the hinge also presents a different orientation between the two species. Moreover, the evaluation of charged and hydrophobic patches in the minimum energy structure highlighted an increased hydrophobic exposed area in cetuximab without Fab glycosylation. The hydrophobic patches include Leu234 and Leu235, residues known to be critical for receptor recognition, suggesting in another way the negative impact of Fab glycosylation on ADCC activation. In summary, this study suggests that: i) Fab glycosylation has an impact on the antibody conformation and on the dynamics of the whole cetuximab; ii) the impact may be negative because of the lower values of CH2 distance, hinge mobility and exposure, and SASA of the residues involved in receptor recognition. These results let hypothesize that also glycosylation pattern could differently modulate the ADCC function, as already mentioned in literature (77), an aspect that will be further investigated in the next future. Our results are supported by the work published by Lippold and colleagues, who applying affinity chromatography and mass spectrometry observed a higher affinity of cetuximab Fc to FcγRIIIa than the intact antibody (78). A similar investigation was performed for FcRn binding residues, showing a decrease of the solvent exposure of these residues, and suggesting a negative impact of this modification also on the FcRn recognition.

Moreover, even if no data are available specifically for cetuximab, recent studies have shown that Fab glycosylation can affect the binding of IgG to human FcRn. This is in line with the data published by Schlothauer et al. who, using IdeS or Plasmin digests that were processed via affinity chromatography, clearly observed an effect of Fab domains on the interaction between Fc and FcRn (45) and also with the study performed by Brinkhaus and colleagues who, via cell membrane - based assays and crystallographic data, showed a negative effect of Fab arms on the FcRn recognition, hypothesizing a steric clash between Fab and the membrane (79). Furthermore, a study carried out by Volkov and colleagues on different Fab glycosylated recombinant antibodies demonstrated a negative impact of Fab glycosylation on the IgG-FcRn interaction in a cellular context. In detail, comparing glycosylated Fab antibodies to non-glycosylated Fab ones an increase in the binding to human FcRn was observed in absence of Fab glycosylation (80). For what concerns the antigen recognition, even if it was not directly investigated herein, our data support the idea that Fab glycans do not influence this aspect, also because their localization in the structure is far away and on the opposite site with respect to CDRs. Accordingly, at least steric hindrance effects are not expected. In conclusion, it can be hypothesized that Fab glycosylation, representing the highest heterogeneity of commercial cetuximab, is a critical aspect to consider during the development of biosimilars in relationship with the potential impact in modulating the ADCC, pharmacokinetics, and, due to conformational constraints, all Fc effector functions. Of note, since this study was entirely developed *in silico*, because of the impossibility to mimic all the *in vivo* physiological conditions, further experimental studies would be required, especially regarding the pharmacokinetics profile and the ADCC activity of cetuximab. On the other hand, the structural complexity due to Fab glycosylation makes the generation of Erbitux^®^ biosimilars very difficult (42) and the results presented in this study can not only provide a rationale to explain this complexity but also pave the way to novel strategies of biotherapeutics development.

## Data availability statement

The raw data supporting the conclusions of this article will be made available by the authors, without undue reservation.

## Author contributions

SS: Writing – original draft, Visualization, Investigation, Formal analysis. DB: Writing – review & editing, Visualization, Formal analysis. OB: Writing – review & editing, Visualization, Formal analysis. MR: Writing – review & editing, Resources, Project administration. UG: Writing – review & editing, Software, Methodology. IE: Writing – review & editing, Supervision, Funding acquisition. FC: Writing – review & editing, Supervision, Conceptualization.
