# Supplementary material for: In silico evaluation of the role of Fab glycosylation in cetuximab antibody dynamics
Source: Front Immunol. 2024 Aug 8;15:1429600. doi: 10.3389/fimmu.2024.1429600 (PMC11342397; doi:10.3389/fimmu.2024.1429600)
Supplement: Supplementary file 1 [file DataSheet_1.docx]

Supplementary Material

***In silico* evaluation of the role of Fab glycosylation in cetuximab antibody dynamics**

**Simona Saporiti^1^, Davide Bianchi^2^, Omar Ben Mariem^2^, Mara Rossi^1^, Uliano Guerrini^2^, Ivano Eberini^3,†^ and Fabio Centola^1,†,*^**

^1^Analytical Excellence and Program Management, Merck Serono S.p.A., Rome, Italy

^2^Dipartimento di Scienze Farmacologiche e Biomolecolari, Università degli Studi di Milano, Via Balzaretti, 9, 20133, Milan, Italy

^3^Dipartimento di Scienze Farmacologiche e Biomolecolari & DSRC, Università degli Studi di Milano, Via Balzaretti, 9, 20133, Milan, Italy

**† Equal contribution**

*** Correspondence:**Corresponding Author
fabio.centola@merckgroup.com


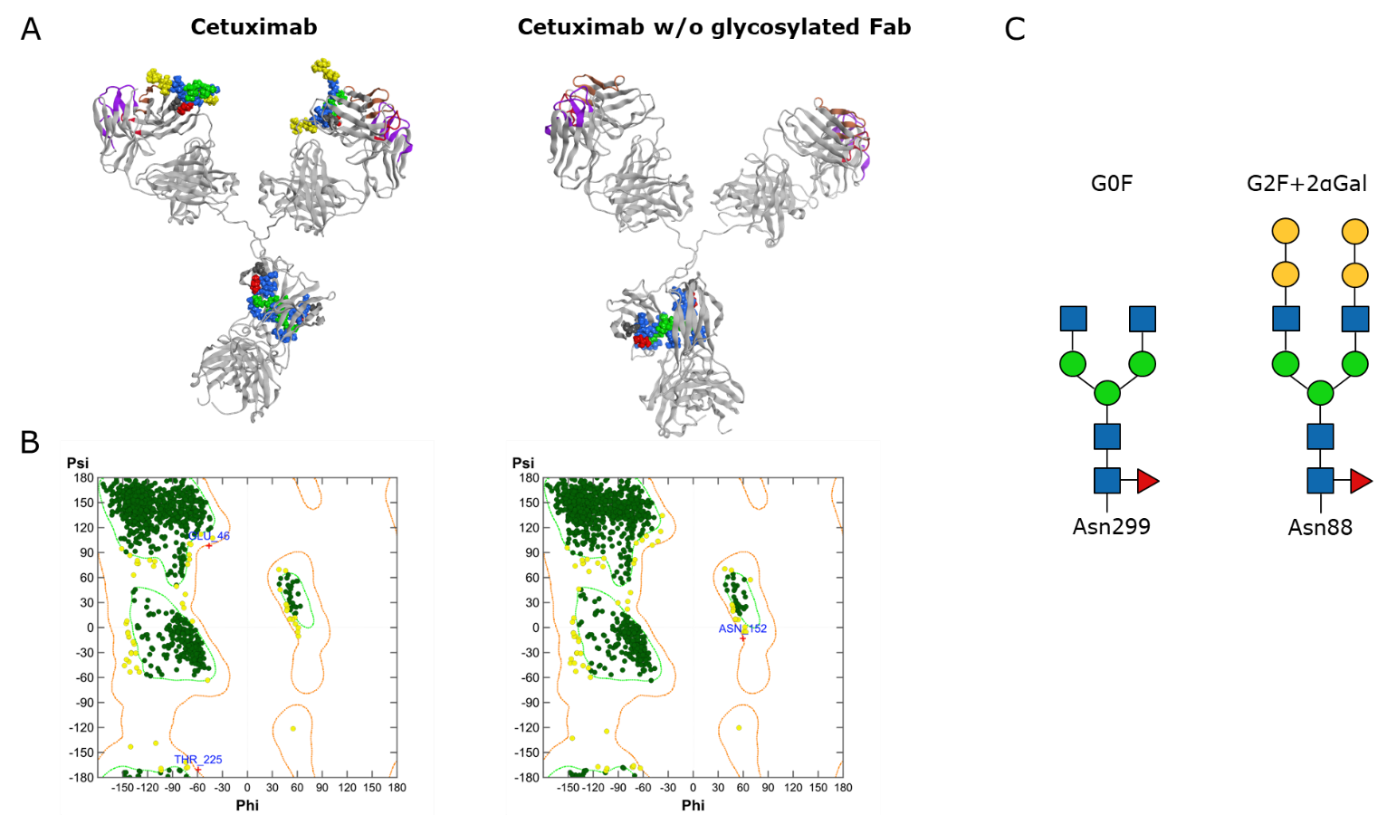


**Supplementary Figure 1: 3D model of cetuximab and cetuximab without Fab glycosylation.** (A) 3D model of cetuximab and cetuximab without Fab glycosylation showed as ribbons. CDR1-3 LC are colored in purple, CDR1-2 HC in maroon and CDR3 HC in red. N-glycans are shown as spheres colored according to SNFG scheme. (B) Ramachandran plot of the two models, red crosses represent outliers. (C) Schematic representation of N-linked glycans according to SNFG.


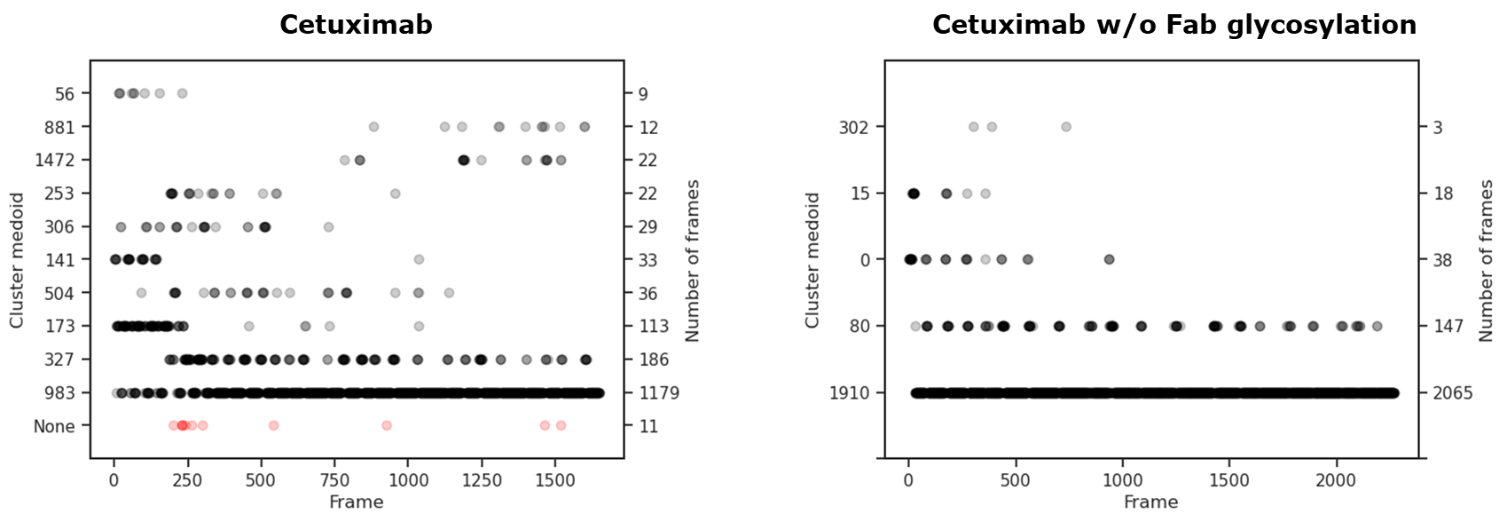


**Supplementary Figure 2: Cluster analysis.** Cluster matrices showing the identified groups in the minimum energy frames with an RMSD-based 6.5 Å threshold. On x-axis, the timeframes, on y-axes, the number of frames included in each cluster and the frame corresponding to the medoid structure.


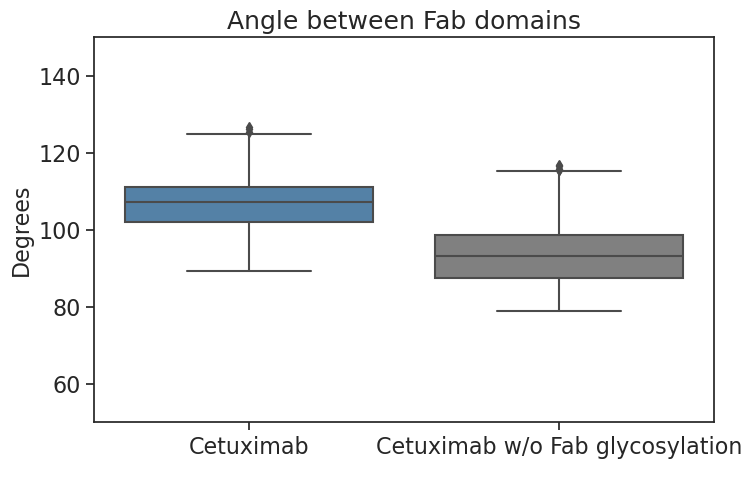


**Supplementary Figure 3: Fab angle distribution.** Boxplot showing the distribution of the values of the angle between Fab domains in cetuximab and cetuximab without Fab glycosylation.


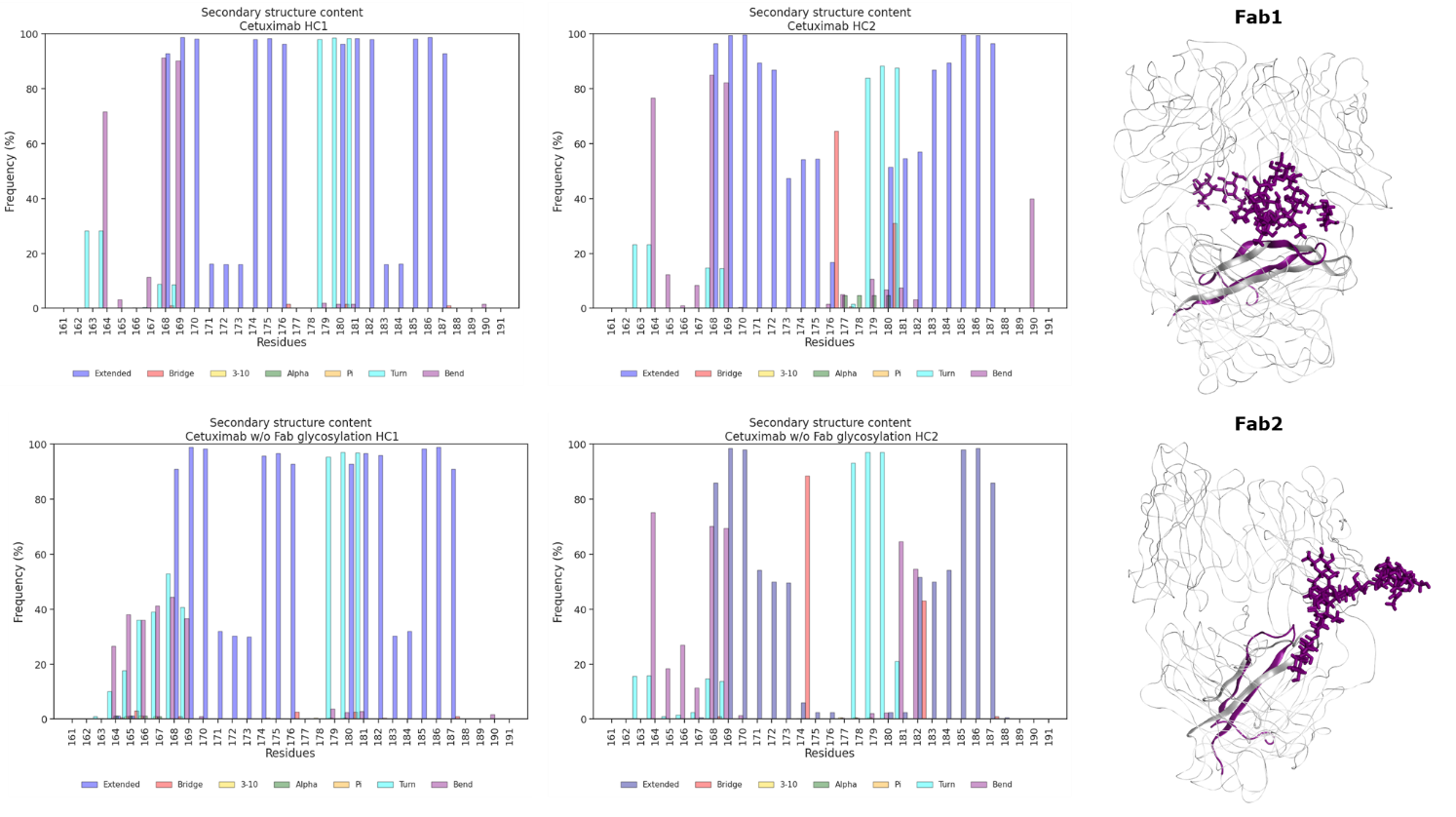


**Supplementary Figure 4: Secondary structure content of HC peptide in cetuximab forms.** On the left, bar plots showing the per residue secondary structure content in the HC peptide (res. 161-191) of cetuximab (top) and cetuximab without Fab glycosylation (bottom). The frequency of each secondary structure type according to DSSP classification is reported in percentage on y-axis. On the right, structural superposition of glycosylated (in dark magenta) and aglycosylated Fab domains (in gray) showing the high similarity in the secondary structure of HC peptides.


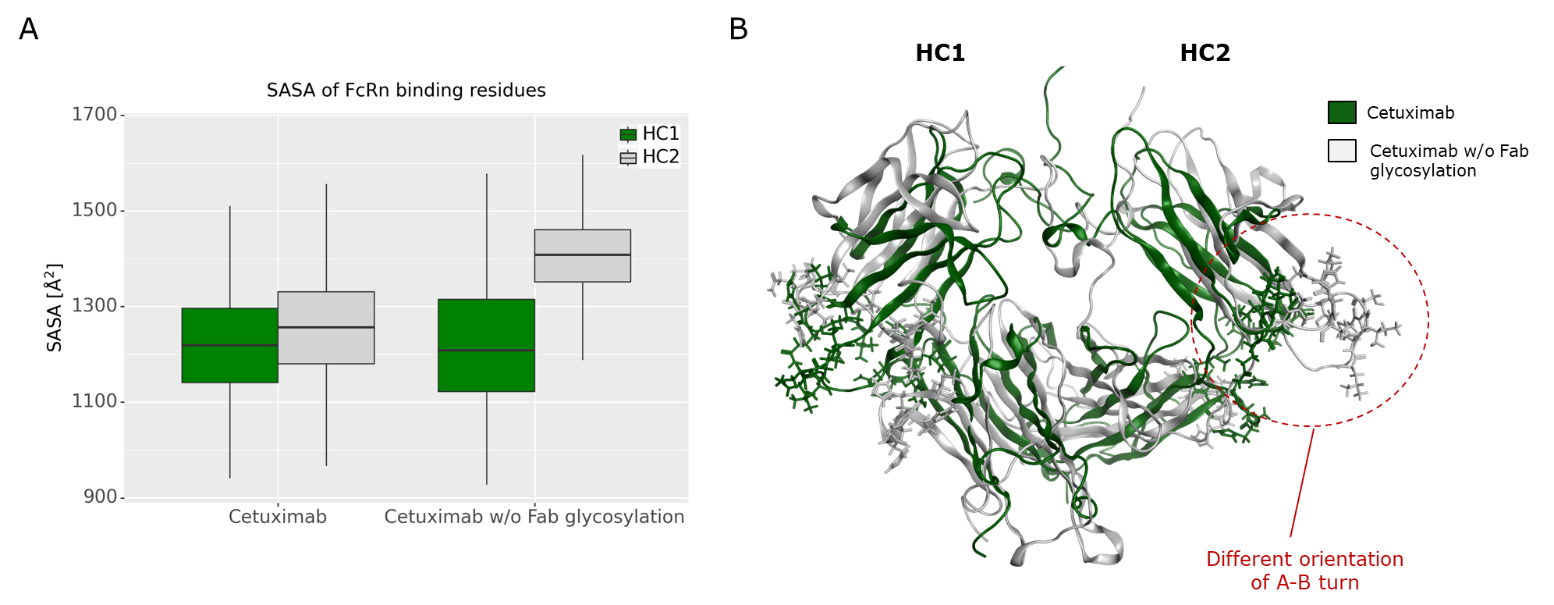


**Supplementary Figure 5: SASA of FcRn binding site residues.** (A) Boxplot showing the distribution of SASA values for FcRn binding residues. (B) Structural localization of FcRn binding sites in the Fc domain of cetuximab (in green) and cetuximab without Fab glycosylation (in gray), with particular emphasis on A-B turn orientation.

**Supplementary Table 1: Accelerated MD parameters in kcal/mol.**

|  | **EthreshD** | **EthreshP** | **alphaD** | **alphaP** |
| --- | --- | --- | --- | --- |
| **Cetuximab** | 19,574 | -1,769,666 | 1,093 | 89,653 |
| **Cetuximab w/o Fab glycosylation** | 18,838 | -1,774,116 | 1,074 | 89,698 |

**Supplementary Table 2: H-bonds interactions between Fab glycans and Fab domains in cetuximab with the associated frequency in the selected energy minimum frames.** In green and bold, those interactions that occur with a frequency up to 10%.

| **Residue** | **Chain** | **Sugar** | **Fab domain** | **Frequency (%)** |
| --- | --- | --- | --- | --- |
| Q1 | HC1 | GAL6 | Fab1 | 1.45 |
| Q3 | HC1 | GAL6 | Fab1 | 1.51 |
| R38 | HC1 | GLC1 | Fab1 | 6.42 |
| **R39** | **LC1** | **GAL6; GAL7** | **Fab1** | **16.34; 10.41** |
| T40 | LC1 | GLC5; GAL6; GAL7 | Fab1 | 4.00; 8.23; 1.15 |
| **S40** | **HC1** | **GLC1** | **Fab1** | **27.06** |
| **N41** | **LC1** | **MAN3**; GLC5; **GLC9** | **Fab1** | **21.13**; 2.60; **39.16** |
| G42 | HC1 | GLC9 | Fab1 | 1.45 |
| **S43** | **LC1** | **GLC5** | **Fab1** | **15.13** |
| K43 | HC1 | GLC1; GAL11 | Fab1 | 3.21; 2.18 |
| **S43** | **LC1** | **GAL6** | **Fab1** | **10.35** |
| Y86 | LC1 | GAL10 | Fab1 | 1.21 |
| S87 | HC2 | GLC1; GAL10 | Fab2 | 1.88; 1.09 |
| **Y87** | **LC1** | **GAL10** | **Fab1** | **29.36** |
| S87 | HC2 | FUC12 | Fab2 | 1.57 |
| **N88** | **HC2** | **GLC1;** GAL10; GAL11; FUC12 | **Fab2** | **22.34;** 3.69; 8.41; 2.18 |
| **N88** | **HC1** | **GLC1** | **Fab1** | **42.62** |
| T90 | HC2 | GAL11 | Fab2 | 3.87 |
| **T90** | **HC1** | GLC1; **FUC12** | **Fab1** | 7.20; **19.25** |
| Y94 | HC1 | GLC5 | Fab1 | 1.27 |
| K103 | LC1 | GLC9; GAL10; GAL11 | Fab1 | 2.36; 8.84; 3.09 |
| Q111 | HC1 | GAL5; GAL6 | Fab1 | 3.15; 3.15 |
| L114 | HC1 | MAN4 | Fab1 | 3.03 |
| T116 | HC1 | MAN4 | Fab1 | 1.57 |
| V117 | HC1 | FUC12 | Fab1 | 1.15 |
| S118 | HC1 | FUC12 | Fab1 | 1.03 |
| A119 | HC2 | FUC12 | Fab2 | 1.45 |
| R142 | LC1 | GLC9; GAL10 | Fab1 | 9.50; 6.60 |
| Q147 | LC2 | GLC5; GAL10; GAL11 | Fab2 | 3.03; 1.27; 1.21 |
| L154 | LC2 | GAL6; GAL7 | Fab2 | 1.03; 1.03 |
| Q155 | LC2 | GLC5; GAL6 | Fab2 | 6.23; 4.54 |
| S156 | LC2 | MAN4; GLC5; GLC9; GAL11 | Fab2 | 1.57; 3.69; 2.91; 1.45 |
| G157 | LC2 | MAN4; GLC9 | Fab2 | 2.54; 2.54 |
| N158 | LC2 | MAN4; GLC5 | Fab2 | 7.02; 1.45 |
| S159 | LC2 | MAN4 | Fab2 | 1.88 |
| Q160 | LC1 | MAN8 | Fab1 | 8.54 |
| Q160 | LC2 | GLC2; GLC5 | Fab2 | 1.33; 1.57 |
| **E161** | **LC1** | **MAN8** | **Fab1** | **64.83** |
| E161 | LC2 | GAL10; FUC12 | Fab2 | 1.39; 1.15 |
| V163 | LC1 | GLC9 | Fab1 | 3.39 |
| T164 | LC1 | GAL7 | Fab1 | 4.42 |
| E165 | LC1 | GAL6; GAL7 | Fab1 | 4.60; 3.21 |
| **D167** | **LC1** | **GAL7** | **Fab1** | **13.62** |
| **S168** | **LC1** | **GAL7** | **Fab1** | **21.55** |
| T171 | HC1 | GAL7 | Fab1 | 9.44 |
| **A174** | **HC1** | MAN3; **MAN4;** GLC5 | **Fab1** | 1.03; **78.93;** 1.03 |
| **L176** | **HC1** | **GLC2; MAN3;** MAN8 | **Fab1** | **43.95; 72.70;** 3.81 |
| S178 | HC2 | GLC9 | Fab2 | 1.39 |
| S179 | HC1 | FUC12 | Fab1 | 5.99 |
| T180 | LC2 | GLC5; GAL11 | Fab2 | 2.48; 2.42 |
| L181 | LC2 | GLC5; GAL11 | Fab2 | 2.06; 1.82 |
| **Y182** | **HC1** | MAN3; **MAN4** | **Fab1** | 3.39; **46.49** |
| S182 | LC2 | GAL6 | Fab2 | 1.21 |

**Statistical significance results**

Statistical significance of the differences between the distributions of Fab-sugars contacts, SASA of hinge, FcγRIIIA and FcRn binding residues, and the angle between Fab domains, was computed using the Student’s t-test and expressing the refusal of the null-hypothesis in terms of *p*-value. Due to the large sample size, as expected all *p*-values are close to 0 and all assessed differences are significant, except the one observed for FcRn binding residues on HC1 (*p*-value = 0.78). Therefore, we also reported the effect size in form of 95% confidence interval of Cohen’s *d*. As usual we considered small effect *d* ~ 0.2, medium effect *d* ~ 0.5, and large effect *d* ~ 0.8 and above. The table below reports these values along with the means and standard deviations of the distributions. Comparing the number of sugar contacts of Fab1 and Fab2 it can be observed a large effect; differences in SASA between the glycosylated and aglycosylated forms show a small to medium effect for HC1 residues interacting with FcγRIIIA and no significant effect for those interacting with FcRn; a large effect in SASA of residues interacting with both receptors in HC2 is observed. Also, the angle between Fab domains shows large effect when comparing the glycosylated and aglycosylated forms.

**Supplementary Table 3: Statistical significance results.** SD = standard deviation; DF = degrees of freedom; CI = confidence interval.

| **Distribution** | **Mean1 ± SD** | **Mean2 ± SD** | **t-test; p-value** | **DF** | **95% CI for Cohen's d** |
| --- | --- | --- | --- | --- | --- |
| **Fab-sugar contacts** | **Fab1** | **Fab2** |  |  |  |
|  | 130 **±** 25 | 44 **±** 20 | 109.3; 0.00 | 3302 | [3.74, 3.87] |
|  | **Glycosylated** | **Aglycosylated** |  | |  |
| SASA Hinge HC1 | 1010 **±** 182 | 953 **±** 184 | 9.74; 0.00 | 3921 | [0.25, 0.38] |
| SASA Hinge HC2 | 1087 **±** 92 | 1178 **±** 82 | -32.38; 0.00 | 3921 | [0.98, 1.11] |
| SASA HC1 FcγRIIIA binding residues | 1084 **±** 127 | 1027 **±** 112 | 14.89; 0.00 | 3921 | [0.42, 0.54] |
| SASA HC2 FcγRIIIA binding residues | 1223 **±** 158 | 1496 **±** 106 | -64.69; 0.00 | 3921 | [2.03, 2.16] |
| Angle between Fab domains | 106 **±** 6 | 93 **±** 7 | 56.04; 0.00 | 3921 | [1.75, 1.88] |
| SASA HC1 FcRn binding residues | 1218 **±** 108 | 1219 **±** 125 | -0.27; 0.78 | 3921 | [-0.05, 0.07] |
| SASA HC2 FcRn binding residues | 1256 **±** 106 | 1405 **±** 83 | -49.1; 0.00 | 3921 | [1.52, 1.65] |
